# Metabolic brain measurements in the newborn: Advances in optical technologies

**DOI:** 10.14814/phy2.14548

**Published:** 2020-09-05

**Authors:** Gemma Bale, Subhabrata Mitra, Ilias Tachtsidis

**Affiliations:** ^1^ Medical Physics and Biomedical Engineering University College London London UK; ^2^ Neonatology, EGA Institute for Women's Health University College London London UK

**Keywords:** biomarkers, cerebral metabolism, intensive care medicine, near‐infrared spectroscopy, neonatal medicine, optical monitoring

## Abstract

Neonatal monitoring in neonatal intensive care is pushing the technological boundaries of newborn brain monitoring in order to improve patient outcome. There is an urgent need of a cot side, real time monitoring for assessment of brain injury severity and neurodevelopmental outcome, in particular for term newborn infants with hypoxic‐ischemic brain injury. This topical review discusses why brain tissue metabolic monitoring is important in this group of infants and introduces the currently used neuromonitoring techniques for metabolic monitoring in the neonatal intensive care unit (NICU). New optical techniques that can monitor changes in brain metabolism together with brain hemodynamics at the cot side are presented. Early studies from these emerging technologies have demonstrated their potential to deliver continuous information regarding cerebral physiological changes in sick newborn infants in real time. The promises of these new tools as well as their potential limitations are discussed.

## Graphical abstract

A summary of the available metabolic neuromonitoring techniques is shown in the abstract diagram.

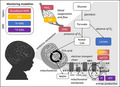

## 1 INTRODUCTION

**Figure 1:**
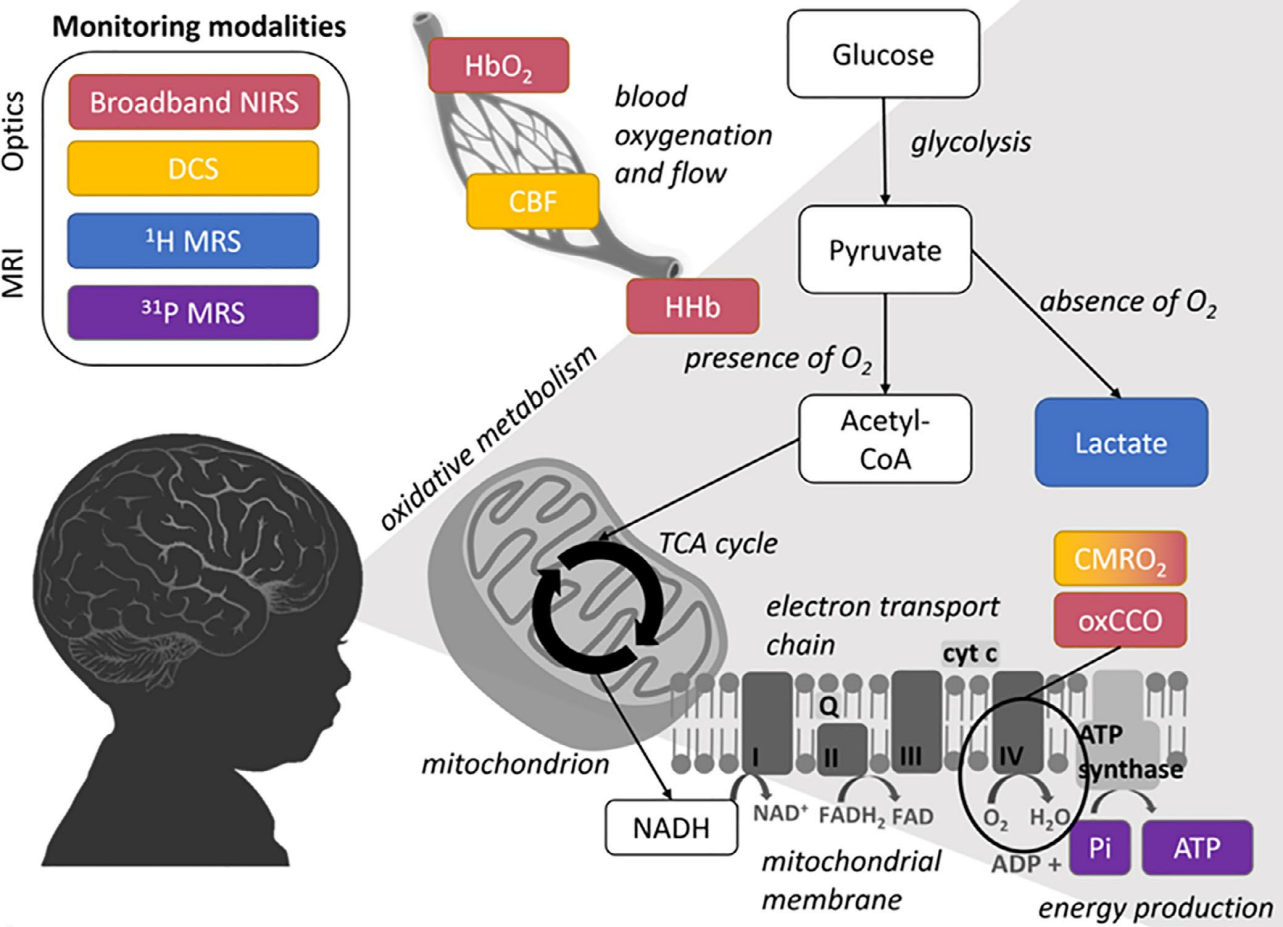
A simplified diagram showing pathways of cerebral oxidative metabolism and associated measurements. Colours link neonatal monitoring modalities to measurement

Neuromonitoring and neuroimaging in the hospital has a key importance for therapeutic decisions and appropriate management, this is especially important in neonatal intensive care unit (NICU). Currently available clinical neuromonitoring solutions in the NICU focus on monitoring brain function using electrical activity (electroencephalography [EEG] with or without amplitude integrated EEG [aEEG]), brain oxygenation (near infrared spectroscopy [NIRS, cerebral oximetry]), and brain structure with cranial ultrasound scan (CrUSS) (Martinello, Hart, Yap, Mitra, & Robertson, 2017). These technologies in isolation or in combination are successful in providing valuable clinical information about the wellbeing of the newborn brain but not optimal for every clinical condition and type of perinatal brain injury (Figure 1).

There is a clear unmet need of a neuromonitoring biomarker in the NICU that can identify brain injury severity in term infants with hypoxic‐ischemic encephalopathy early in life and predict neurodevelopmental outcome. The majority of perinatal brain injury in term newborn infants results from hypoxic ischemic encephalopathy (HIE). HIE is a significant cause of global mortality and disability in children. It occurs mostly around the time of birth, however the pathophysiology evolves over a prolonged period after the initial insult (Fatemi, Wilson, & Johnston, 2009). A significant change in brain metabolism has been identified in HIE using magnetic resonance spectroscopy (MRS) studies that further identifies the need for a brain tissue metabolic indicator of injury severity at the cot side.

**Figure 2:**
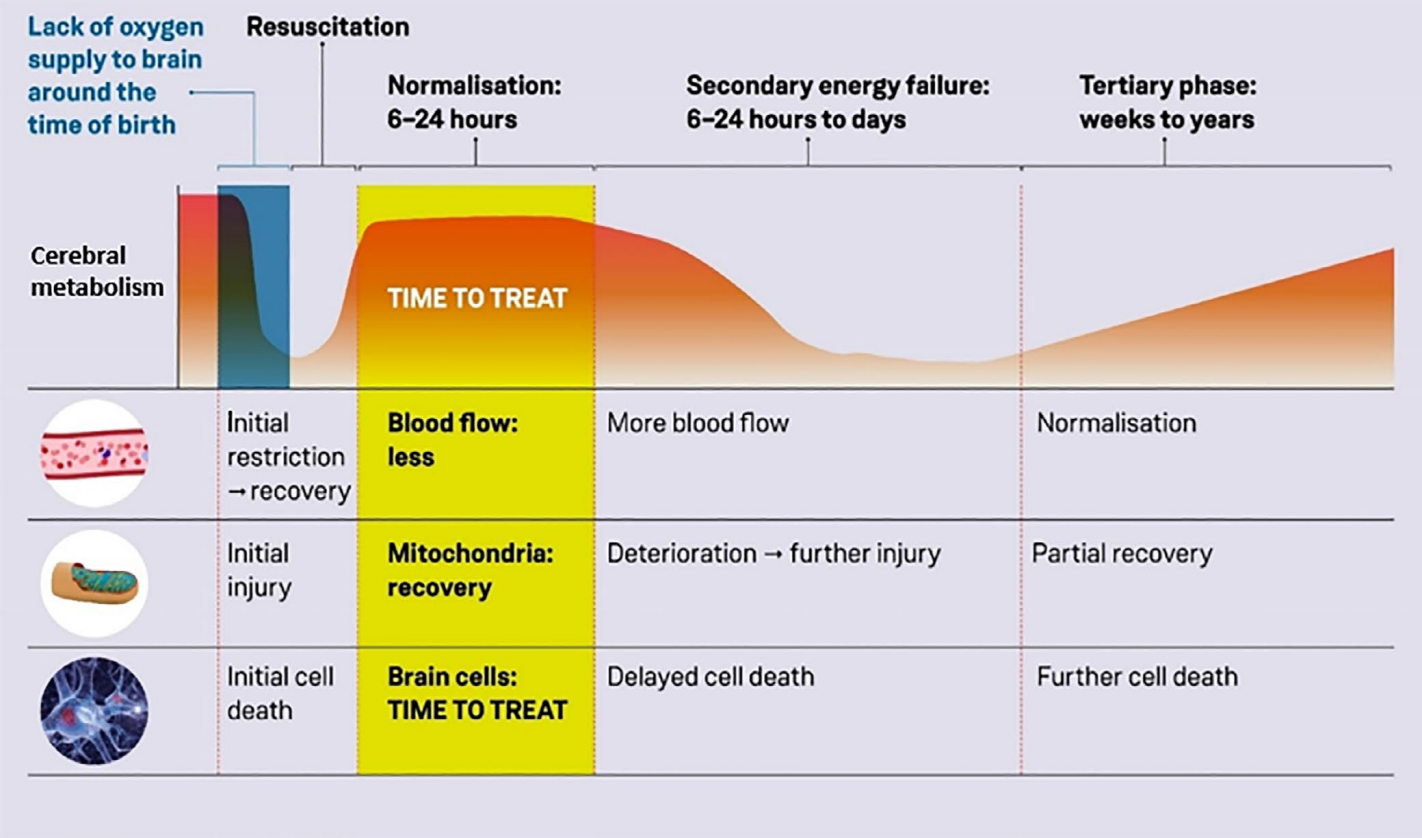
Progression of theorised changes in cerebral energy metabolism during HIE

There is an evolution of a delayed cascade of molecular events triggered by the initial insult (Fatemi et al., 2009). Phosphorus (^31^P) and proton (^1^H) MRS studies have confirmed a significant and prolonged changes in brain metabolism following HIE (Azzopardi et al., 1989; Mitra et al., 2018a). A biphasic pattern of cerebral energy failure following HIE was demonstrated using on ^31^P and ^1^H MRS studies; the first phase occurs during the insult (primary energy failure), which is followed by re‐perfusion and a period of cerebral recovery. After a latent period of up to 24 hr, a secondary energy failure may occur which can cause severe cell death and injury with an increase in lactic acid (Lac), produced in anaerobic metabolism, and a reduction of N‐acetyl‐aspartate (NAA), indicating neuronal loss, over the first few days after initial insult. Figure 2 shows a schematic of this process. During this latent phase there is the potential for clinical intervention, however it is difficult to assess the severity of the initial injury; this is the concept of a brief ‘therapeutic window’ between the time of insult and the occurrence of cell death due to a cascade of molecular events.

Therefore, it is vital to monitor metabolic processes during the first hours of HIE, in order to assess the injury severity and have an opportunity to intervene with adjunct neuroprotective therapies, emerging on the horizon (Hassell, Ezzati, Alonso‐Alconada, Hausenloy, & Robertson, 2015). Using improved neuromonitoring to target therapies to patients, that is, the concept of “personalized medicine,” may improve the efficacy of those potential therapies if they are directed at patients which will most benefit from them.

Current options for measurements of brain metabolism in the NICU are described below along with their benefits and issues; followed by a review of the recent state‐of‐the‐art optical techniques which have the potential to provide a cot side brain biomarker that could revolutionize neonatal brain monitoring and improve outcome. In particular, we describe advances in diffuse optical methods, near‐infrared spectroscopy and diffuse correlation spectroscopy, and discuss their clinical potential.

## 2 NEUROLOGICAL MONITORING: CURRENT STATUS

In intensive care units, systemic monitoring is an essential part of routine monitoring in any sick newborn infants as the optimization of patient's hemodynamic status, ventilation, temperature, nutrition, and metabolism is the key to improve patients' survival. Current brain monitoring used in the routine care of neurologically compromised infants in the neonatal intensive care unit can be split into two categories: “continuous” and “snapshot” measurements. Continuous measurements include EEG/aEEG and NIRS cerebral oximetry, whereas MRI and MRS are limited to providing “snapshots” of the cerebral function.

There are also some other metabolic measurement methods, such as fluorodeoxyglucose (FDG)‐positron emission tomography (PET) which can provide images of cerebral metabolic rate of glucose consumption (Shi et al., 2012) (although recent evidence suggests that other pathways may contribute to the FDG‐PET signal (Cossu et al., 2019)), that are unlikely to be adopted into regular NICU practices because of safety concerns relating to the radiation exposure in neonates.

There are also structural cerebral monitoring devices which can monitor the evolution of injury. Cranial ultrasound (CrUSS) is a bedside tool for assessment of cerebral structures and can be repeated regularly as the hypoxic ischemic injury evolves with time, but early examination can be normal even in moderate to severe HIE (Martinello et al., 2017).

### 2.1 Electroencephalogram (EEG) and amplitude‐integrated electroencephalogram (aEEG)

Electroencephalography provides an excellent opportunity to continuously monitor brain function starting from soon after birth, but use of full neonatal EEG needs a highly specialized neurophysiological support for application and reporting along with the need for a team of well‐trained clinicians and nurses for continuous monitoring. EEG shows a typical pattern of changes in infants with moderate to severe encephalopathy, who respond well to therapeutic hypothermia treatment. The initial EEG activity remains suppressed for several hours with flat traces before bursts of activity start to appear with or without seizures. Depending on the severity of injury and response to treatment, the EEG becomes more and more continuous as time progresses. The prognostic value of EEG in HIE has been investigated in several studies (Pressler, Boylan, Morton, Binnie, & Rennie, 2001). In a video‐EEG polygraphic study, Pressler et al. noted that early recovery of EEG within 8h is associated with a favorable outcome and is related to poor outcome if the traces remain discontinuous or grossly abnormal beyond 8–12 hr following HI.

**Figure 3:**
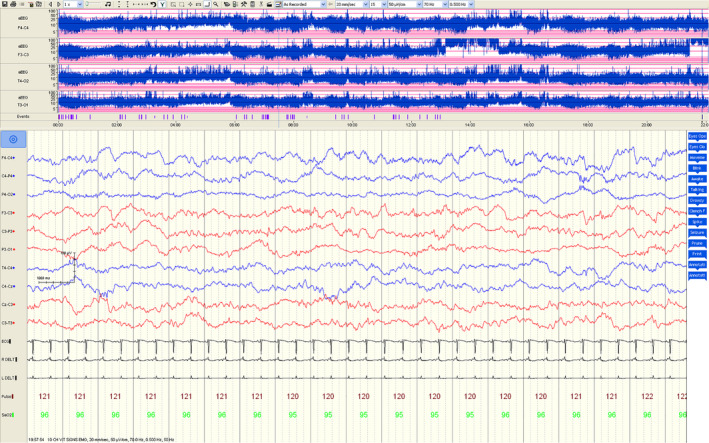
An example of a normal aEEG in the upper panel along with EEG in a term infant

Amplitude integrated EEG (aEEG) is the most commonly used neuromonitoring tool in the NICU. The EEG signal from one or two channels is amplified, passed through asymmetrical band‐passed filter at 2–15 Hz (to minimize artifacts), rectified (negative values are changed to positive), smoothed and time compressed. The output is displayed on a semi logarithmic scale (see example of data in Figure 3). Due to its simplicity, aEEG has become very popular in neonatal brain monitoring. aEEG traces are classified based on either voltage or pattern recognition (Thoresen, Hellstrom‐Westas, Liu, & de Vries, 2010). The value of the background pattern in neurodevelopmental outcome prediction following HIE has been extensively investigated using aEEG. Although a poor background pattern persisting beyond 12–24 hr has been documented with poor outcome in earlier studies, recent studies on infants undergoing therapeutic hypothermia revealed that hypothermia can cause delay in return of the normal background pattern up to 48 hr (Thoresen et al., 2010). EEG and aEEG had high sensitivity and specificity (EEG: sensitivity 0.92 [0.66–0.99]; specificity 0.83 [0.64–0.93] and aEEG: sensitivity 0.93, [95% confidence interval 0.78–0.98]; specificity 0.90 [0.60–0.98]) for early (within 6 hr of age) prognostication after perinatal asphyxia in the pre‐cooling era (Van Laerhoven, De Haan, Offringa, Post, & Van Der Lee, 2013). Despite its high prognostic value at 6 hr, there is still a need for an indicator of the progression of HIE and response to treatment in the following days.

### 2.2 Near‐infrared spectroscopy (NIRS) cerebral oximetry

In 1977, Franz Jöbsis discovered an optical window in the near‐infrared (NIR) spectrum; light in the region of 650‐1000 nm travels through tissue and therefore it is possible to interrogate absorbing compounds within the tissue (Bale, Elwell, & Tachtsidis, 2016). The strongest absorbers in this region are also clinically interesting; hemoglobin in both oxygenated and deoxygenated (HbO_2_ and HHb) forms, and the oxidation state of cytochrome c oxidase (oxCCO), a mitochondrial marker of metabolism.

NIRS systems are relatively simple: they essentially consist of a light source, which illuminates tissue, and a detector, to record light that has travelled through and exits the tissue (the light can either travel through back‐reflection or transmission across the tissue). The monitored changes in light attenuation from the tissue are related to the changes in concentration of the absorbers within the tissue. It is possible to calculate these changes by comparing them to the known absorption spectra of the absorbers, this is done using the modified Beer‐Lambert law. This technique allows real insight into the hemodynamic and metabolic changes within tissue, such as the brain and muscle, in a non‐invasive, non‐ionizing, relatively inexpensive, portable manner. Instrumentation that uses the NIRS concept and quantifies an absolute percentage number of mixed arterial venous oxygenation in the brain is called cerebral oximetry.

NIRS cerebral oximetry uses a multi‐distance method to monitor tissue saturation (StO_2_) during HIE (Wolf, Naulaers, van Bel, Kleiser, & Greisen, 2012). This technique is portable, easy to apply and gives a continuous measurement. Several studies have found that infants with an adverse outcome after HIE have higher StO_2_ (Ancora et al., 2013; Nakamura et al., 2015) or increasing StO_2_ (Toet, Lemmers, vanSchelv en, & vanBel, 2006) compared to those with normal outcome; some studies saw lower StO_2_in those with adverse outcome (Massaro, Bouyssi‐Kobar, Chang, Vezina, du Plessis, et al., 2013; Wintermark, Hansen, Warfield, Dukhovny, & Soul, 2014) or in infants with HIE compared to age‐matched healthy controls (Huang et al., 2004) (note that infants were not treated with therapeutic hypothermia in this study); and two studies found no significant difference between infants with adverse and normal outcomes (Shellhaas, Kushwaha, Plegue, Selewski, & Barks, 2015) or in infants with HIE compared to age‐matched healthy controls (Ellen Grant et al., 2009).

If cerebral oximetry is combined with pulse oximetry, the fractional tissue oxygen extraction fraction (FTOE), a more direct marker of oxygen metabolism, can be derived (Naulaers et al., 2007). FTOE presents the balance between oxygen delivery and oxygen consumption: FTOE = StO_2_ – SpO_2_, where SpO_2_ is arterial saturation measured by pulse oximetry and is a surrogate for brain arterial saturation. A rise in FTOE indicates an increased oxygen consumption by tissue relative to oxygen delivery. FTOE has been shown to decrease from 24 hr of age onward in a HIE group with adverse outcome group as compared with the favorable outcome group in both the pre‐cooling era and in the cooling era (Lemmers et al., 2013; Toet, Lemmers, vanSchelven, & vanBel, 2006).

A recent multi‐center clinical trial assessed ability of cerebral oximeters to impact preterm intensive care. The trial demonstrated that informing care with oximetry could reduce the burden of hypoxia but did not see any link between outcome of patients and oximetry (Plomgaard et al., 2019). International efforts to establish optical monitoring techniques in the NICU should be used as an example for the introduction and acceptance of newer techniques (such as those described in Section 3). A systemic review of cerebral oximetry in neonatal care which examines this technique in more depth has been recently published (Mitra, Bale, Meek, Tachtsidis, & Robertson, 2020).

The work in NIRS cerebral oximetry and HIE suggests that cerebral oxygenation is not related to injury severity in a consistent way and this may be due to the heterogeneity of HIE injury, patients and treatment, and also the different pathophysiology that abnormal StO_2_ can represent.

### 2.3 Magnetic resonance imaging and spectroscopy (MRI/MRS)

Magnetic resonance imaging (MRI) and spectroscopy (MRS) have helped us to understand the pathophysiology of evolving injury in HIE and have become an essential tool for assessment of injury severity following HIE and for outcome prediction (Agut et al., 2014). MRI of the brain is the imaging modality of choice following HIE and, together with MRS, is used for assessment of injury severity and future prognostication (Alderliesten et al., 2017).

**Figure 4:**
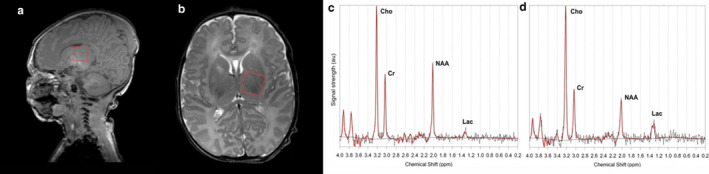
Position of the thalamic 1H magnetic resonance spectroscopy voxel for spectral acquisition (sagittal A and axial B) from a neonate with normal outcome (Lac/NAA 0.19) (C) and a neonate with poor outcome (Lac/NAA 0.62) (D) after HT for NE. Cho, choline; Cr, creatine; HT, hypothermia; Lac, lactate; NE, neonatal encephalopathy; NAA, N‐acetylaspartate. Reproduced with permission from Mitra et al. (2018b)

^1^H MRS is being increasingly used in neuroimaging following HIE and deep grey matter Lac/NAA is the most accurate quantitative MR biomarker for prediction of abnormal neurodevelopmental outcome (Mitra et al., 2018a) (see example of measurement in Figure 4). NAA is a marker of neuronal/axonal density and viability while cerebral lactate indicates failed oxidative phosphorylation and/or increased anaerobic glycolysis.

Phosphorus‐MRS is useful for quantitation of high‐energy phosphates, which usually are normal in the first hours after birth and begin to decline later in the first 24 hr, apparently with onset of secondary energy failure (Azzopardi et al., 1989), yet the technical challenges of ^31^P MRS means that it is not clinically used yet.

Along with T1 and T2 weighted sequences, newer conventional MRI techniques like diffusion weighted imaging (DWI) have helped us to diagnose lesions earlier than conventional images. Arterial spin labeling (ASL) MRI can evaluate cerebral perfusion in encephalopathic newborns. Using ASL, cerebral blood flow (CBF) was noted to be increased in the second week in infants following HIE, particularly in the basal ganglia and thalamic area (Massaro, Bouyssi‐Kobar, Chang, Vezina, duPlessis, et al., 2013). De Vis et al. also found increased perfusion in infants with adverse outcome following HIE (De Vis et al., 2015). Global cerebral oxygen consumption (CMRO_2_) can also be assessed in neonates using a new technique, T2 relaxation under spin tagging (TRUST) (Shetty et al., 2019). Techniques such as perfusion weighted imaging MRI have been shown to identify hyperperfusion in both watershed and basal ganglia pattern injuries (Wintermark, Moessinger, Gudinchet, & Meuli, 2008).

It should be emphasized that the MRI scan is only performed at one time point during intensive care and gives “snapshot” information rather than continuous, evolving information regarding the pathophysiological changes in brain.

The current metabolic monitoring in the NICU is limited by the difficulty of the technological challenges, feasibility of performing the measurements at the cot side, real‐time, non‐invasively and be able to apply them soon after birth. While MRI techniques are able to give an in‐depth view of cerebral metabolism and perfusion alongside detailed anatomy, they can only be performed as “snapshots” of the patients status and cannot give continuous information throughout treatment. Table 1 summarizes the key techniques.

**Table 1:** Summary of measurements currently in use to monitor cerebral metabolism in the NICU

Technique	Measurement	Clinical Acceptance	Time of Measurement	Location of Measurement
EEG	Neuronal electrical activity	Uncommon	Continuous, can be started immediately after birth	Cot side, portable
aEEG	Neuronal electrical activity	Common	Continuous, can be started immediately after birth	Cot side, portable
NIRS cerebral oximetry	StO_2_, FTOE (if combined with pulse oximetry)	Uncommon	Continuous, can be started immediately after birth	Cot side, portable
MRI TRUST	CMRO_2_	Research	Snapshot, typically after treatment	MRI scanner
^1^H MRS	Lactate/NAA peak area ratio	Infrequent	Snapshot, typically after completion of treatment	MRI scanner

## 3 NEONATAL NEUROLOGICAL MONITORING: ADVANCES IN DIFFUSE OPTICS

Future and currently in‐trial optical methods have the potential to provide continuous, non‐invasive, cot side measurements of oxygenation, hemodynamics, perfusion, and metabolism. Here we present the latest updates from the field of biomedical optics.

### 3.1 Broadband near infrared spectroscopy (bNIRS)

Advanced NIRS methods have been used in research to obtain additional information about the brain. Using multiple wavelengths of light (broadband), bNIRS can additionally monitor another absorber of light, cytochrome c oxidase (CCO), and provide information regarding oxidative metabolism. CCO is the terminal electron acceptor in the electron transport chain, the final stage of oxidative metabolism. The CCO signal does not arise from changes in concentration of CCO itself (the concentration of CCO is stable over a measurement period) but rather from changes in its oxidation state. A unique copper dimer in the enzyme has an absorption peak around 835 nm in its oxidized form, but not in its reduced state. Therefore, bNIRS is able to resolve changes in the oxidation state using the difference spectrum between the oxidized and reduced species to obtain an indicator of the changes in the CCO redox state. A change in the redox state represents a change in oxidative cellular metabolism which is dependent on tissue oxygen and glucose availability, along with other factors such as cellular injury. Monitoring the CCO redox state changes gives an indication of changes in mitochondrial oxidative metabolism at a cellular level. NIRS measurements of metabolism have the potential to yield crucial information about cerebral metabolism and tissue oxygenation at the baby's cot side.

**Figure 5:**
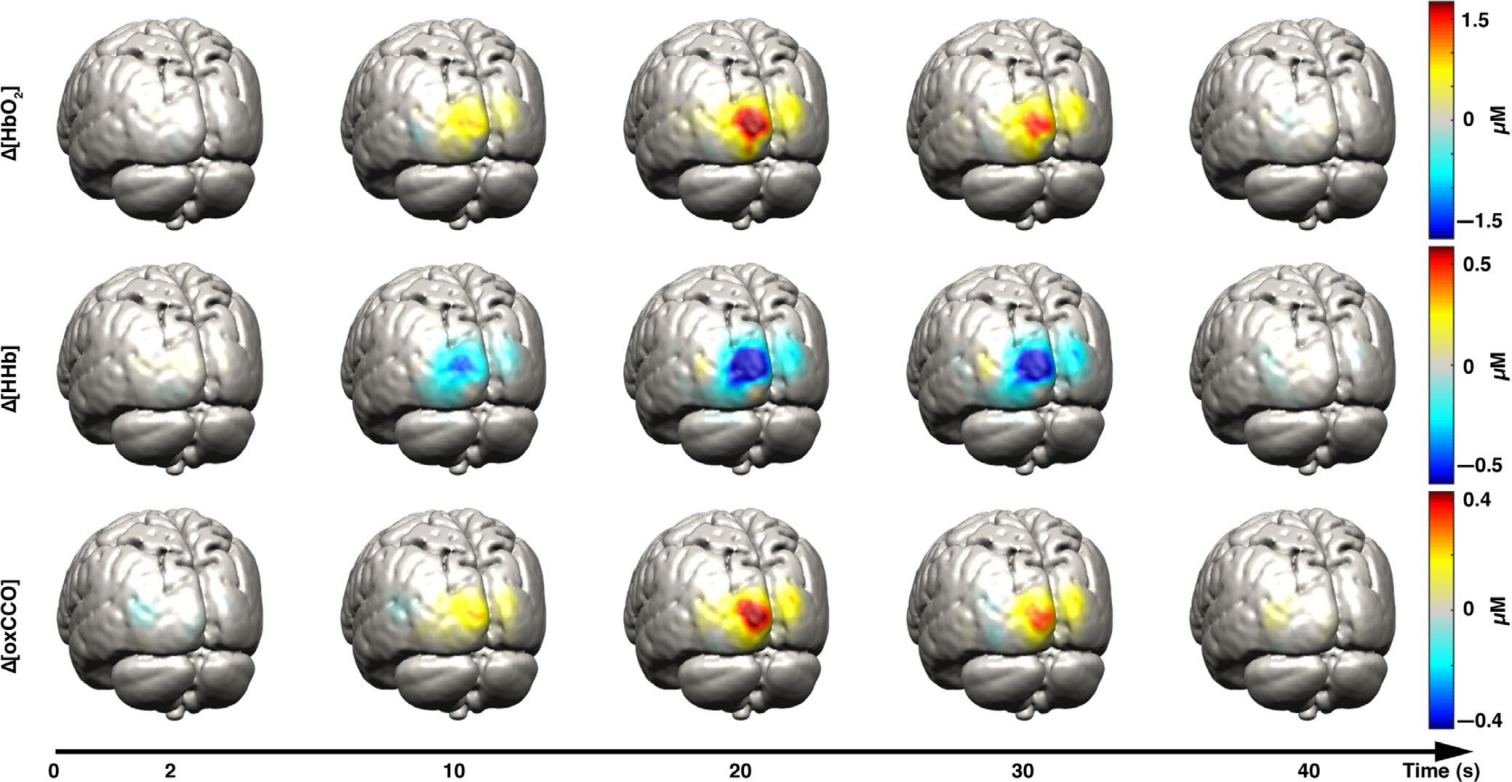
Examples of reconstructed HbO2 (first row), HHb (second row), and oxCCO (third row) images on the grey matter surface mesh at five different time points during visual stimulus presentation. The selected time points are 2, 10, 20, 30, and 40 s after stimulus presentation. Note the increase in HbO2 and oxCCO, with corresponding decrease in HHb as a response to functional stimulation. Reproduced with permission from Brigadoi et al. (2017)

A novel bNIRS instrument (8 channels, 770‐906 nm) was developed specifically for neonatal monitoring of cerebral CCO (Bale, Mitra, Meek, Robertson, & Tachtsidis, 2014). We have demonstrated (a) that relationship of brain oxygenation and CCO during spontaneous desaturation events can be indicative of the severity of the brain injury (Bale et al., 2019) (Figure 5) (b) the association between the brain tissue CCO and systemic physiology fluctuations, represented as ‘metabolic reactivity’, is highly correlated in infants with severe HIE (Mitra et al., 2019); and (c) the relationship between oxygenation and metabolism during rewarming after therapeutic hypothermia was depended on the severity of injury (Mitra, Bale, Meek, Uria, et al., 2016). In addition, we have demonstrated that the continuous metabolic measurement of CCO provides unique information regarding brain health during neonatal stroke (Mitra, Bale, Meek, Mathieson, et al., 2016) and neonatal seizures (Mitra, Bale, Mathieson, et al., 2016).

This work is supported by preclinical studies of HIE. In a study combining bNIRS and ^31^P MRS in 22 piglets we showed that following HI the recovery of oxCCO was correlated with the recovery of nucleotide‐triphosphate (NTP)/total exchangeable phosphate pool (epp) and outcome (Bainbridge et al., 2013). Our results suggested that if NTP/epp and oxCCO recovery is not above a threshold following HI, unfavorable outcome is likely (Kaynezhad et al., 2019). In addition, we observed that the measurements of the changes in brain tissue hemoglobin difference (HbD = HbO_2_ − HHb) which is indicative of brain oxygenation show a poor correlation with NTP/epp and outcome.

Broadband NIRS systems were general expensive, bulky and not easily recognized as clinical instruments limiting their usage and application. These limitations have been largely dependent in the lack of availability of compact light sources that can deliver enough power through a fiber optic cable and the absence of miniaturized photonic detector systems that have enough sensitivity to monitor low light levels. However, recent development in photonic devices and components allow us to overcome these issues and now miniaturized bNIRS systems that use adapted micro‐spectrometers and compact light sources have made the technology more portable, inexpensive and robust (Siddiqui, Kaynezhad, Tachtsidis, Johnson, & Elwell, 2018; Diop, Wright, Toronov, Lee, & St Lawrence, 2014) making them more appropriate for the NICU.

**Figure 6:**
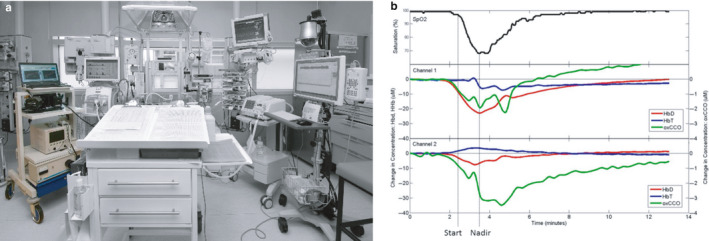
(a) Photo of bNIRS and NIRS monitoring (far left, in colour) brain metabolism in the NICU. (b) Example of continuous NIRS data with pulse oximetry (top) showing bilateral responses in cerebral oxygenation and metabolism to arterial desaturation (lower 2 graphs). The metabolic response to desaturation was found to be predictive of brain injury severity; poor outcome is predicted when there is a coupling of metabolism and oxygenation, as seen in this example (Bale et al., 2019)

To produce an image the physiological processes within the brain, NIRS can be expanded with multiple channels to build up topographic or tomographic images; this is called diffuse optical tomomography/topography (DOT) and has been applied in neonates at the cot side (Ferradal et al., 2016; Cooper et al., 2011). Recent advances in bNIRS have produced the first images of oxCCO with DOT (see Figure 6) (Brigadoi et al., 2017). The reconstructed images show that oxCCO gives a more localized signal than the hemoglobins; the oxCCO signal originates from the neurons and other brain cells rather than the surrounding vasculature. This has the potential to move into the NICU for full cortical metabolic imaging which would help identify metabolic abnormality in specific brain legions after HIE. This could be deployed in assessment of seizures or use in functional paradigms.

### 3.2 Diffuse correlation spectroscopy (DCS)

Diffuse correlation spectroscopy is the measurement of the diffusing temporal field autocorrelation function to obtain an index of cerebral blood flow (CBF_i_ or BFI). DCS detects blood flow by quantifying temporal fluctuations of light fields emerging from the tissue surface. Typically, these light fields are generated by illuminating the brain surface with NIR laser light; some of the NIR light propagates through the scalp and skull and into the brain where it is scattered by moving red blood cells in tissue vasculature before emerging from the tissue surface. This “dynamic” scattering from moving cells causes the detected intensity to temporally fluctuate, and the time scale of these fluctuations is quantified by the intensity temporal autocorrelation function of the collected light, giving an index of CBF called BFI or CBF_i_ (Gonzalez et al., 2014). The change from the initial CBF_i_ is called rCBF (relative change in CBF over time) and rCBF has been shown to correlate with other measures of changes in CBF from other modalities (i.e., fluorescence, bolus‐tracking NIRS, and ASL‐MRI) (Gonzalez et al., 2014).

Neonates are an ideal population to study with DCS, because they have thinner extracerebral layers than adults. Thus, in this population the community has observed higher SNR, excellent reproducibility of CBF_i_, and greater sensitivity to cortical tissue as compared to adults. Neonatal experimental results encompass both episodic monitoring as well as periodic quantification of absolute CBFi. Episodic monitoring has been used to quantify the response to hypercapnia, postural manipulation, drug delivery, surgical intervention, therapeutics, and functional activation. To retrieve an index of CMRO_2_, CMRO_2i_ can be calculated directly from CBFi when it is combined with StO_2_ from NIRS cerebral oximetry and arterial saturation from pulse oximetry (Dehaes et al., 2014).

Dehaes et al. have observed substantial decreases in CBFi and CMRO_2i_ in hypoxic ischemic injured neonates under therapeutic hypothermia (Dehaes et al., 2014). The ability to measure CMRO_2_ during therapeutic hypothermia and assess its dynamic may help to individually optimize and guide this therapy. Roche‐Labarbe et al. recently demonstrated the feasibility of DCS functional studies in infants (Roche‐Labarbe et al., 2013). Periodic measures of CBF_i_ have been used to investigate changes in cerebral hemodynamics over the first month of development in term and preterm infants (Roche‐Labarbe et al., 2012) as well as regional and hemispheric differences in cortical flow (Lin et al., 2012). Furthermore, the works by Roche‐Labarbe et al. (2012) and Dehaes et al. (Dehaes et al., 2014), in particular, highlight the relative insensitivity of StO_2_ alone, that is, the standard parameter provided by commercially available stand‐alone NIRS devices, as a measure of brain health. Furthermore, a combination of NIRS and DCS allows us to examine neurovascular coupling in the immature (preterm) cortex (Nourhashemi, Kongolo, Mahmoudzadeh, Goudjil, & Wallois, 2017). These works demonstrate the obvious advantage that DCS adds in providing a more complete picture of neonatal development through quantification of CBF and CMRO_2_ (Gonzalez et al., 2014). Recently an integrated time resolved NIRS and DCS device has been developed specifically to monitor the preterm brain in the NICU, the BabyLux (Giovannella, 2019).

### 3.3 Discussion of novel optical techniques to measure metabolism

**Figure 7:**
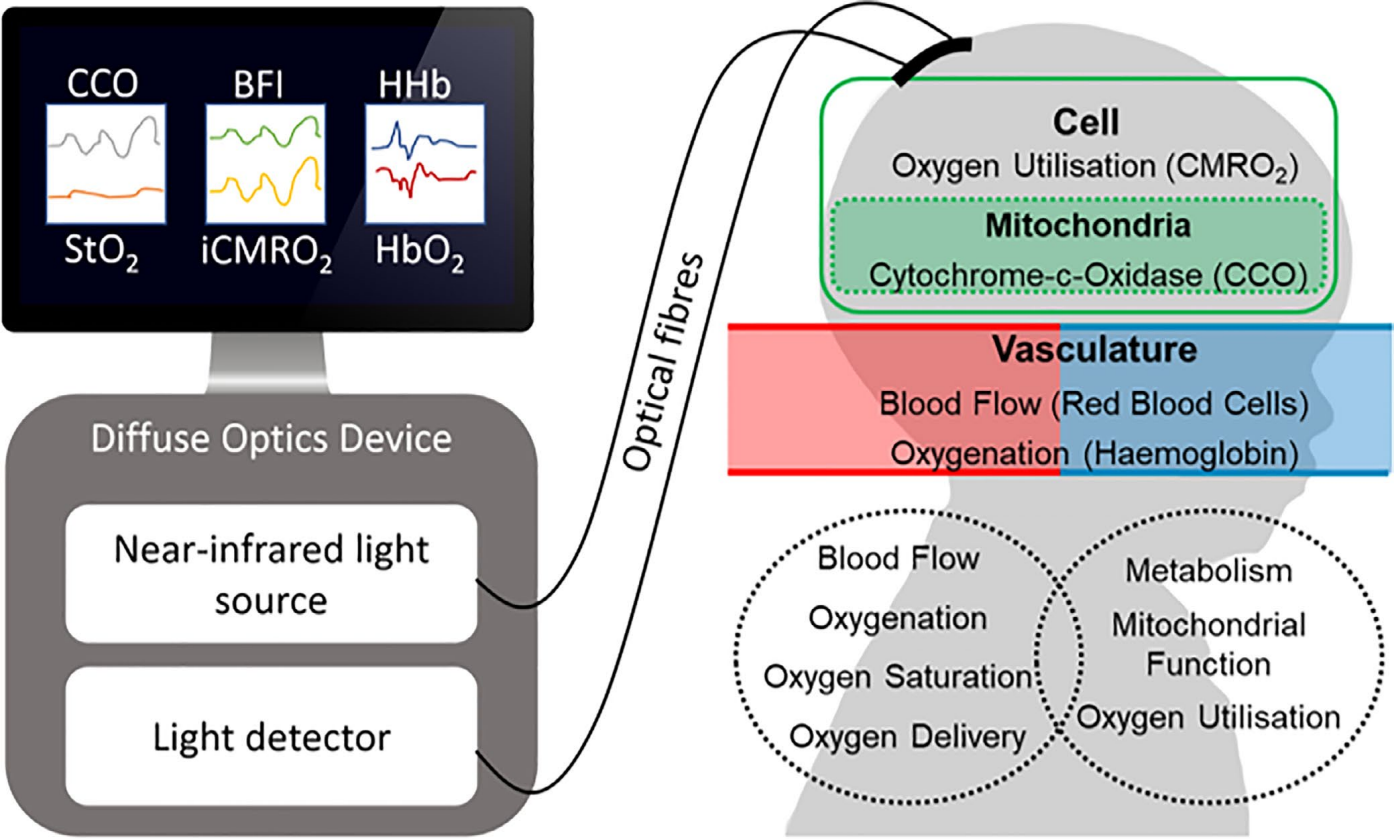
Diagram showing typical set up of diffuse optics with measurements and corresponding physiology. The diffuse optics device could include NIRS cerebral oximetry, NIRS, bNIRS, DOT or DCS. (See Table 2 for details on the measures from each device.)

The above discussed research demonstrates that optical monitoring in the NICU is feasible and has the capacity to monitor tissue oxygenation, perfusion, and metabolism as well as identifying optimum autoregulatory pressures. Figure 7 summarizes the measurements capable with diffuse optics and Table 2 breaks down the techniques.

**Table 2:** Key optical techniques for cot side monitoring in the NICU. All techniques are in research stage have not been clinically applied

Technique	Measurement	Time of Measurement	Location of Measurement
NIRS	HbO_2_, HHb	Continuous, immediately after birth	Cot side, portable
bNIRS	oxCCO, HbO_2_, HHb	Continuous, immediately after birth	Cot side, portable
DCS	CBFi, iCMRO_2_ (if combined with NIRS and pulse oximetry)	Continuous, immediately after birth	Cot side, portable

While these techniques can measure cellular/oxygen metabolism continuously at the cot side, there are some limitations that might hinder their applicability in clinical care. Regarding bNIRS, the relative changes of oxCCO needs to be assessed with respect to other systemic or cerebral changes; hence allowing a better interpretation of the physiology as recently demonstrated by our group (Mitra et al., 2019; Bale et al., 2018). However, in preclinical studies when the initial hypoxic‐ischemic insult is recorded this measurement has demonstrated a great potential to predict the MRS metabolic markers and assess injury severity (Bainbridge et al., 2013; Kaynezhad et al., 2019). DCS can provide an absolute measurement of blood flow and therefore CMRO_2_, it also suffers from limitations that are intrinsic to all optical monitoring such as motion artifacts and tissue inhomogeneities. Trend monitoring offers a plethora of information of the brain physiology that is often missed and not appreciated due to complexities in interpretation; this is a limitation of the technology, and how data is presented; researchers are working towards rectifying this. However, continuous, dynamic monitoring techniques provide real‐time information on the cerebral and hemodynamic status of the neonate and can serve as an important adjunct to patient care (Garvey, Kooi, Smith, & Dempsey, 2018). Methods must be developed to robustly, reproducibly and accurately identify HIE injury and this can only be done by engineers and clinicians working together.

## 4 CONCLUDING REMARKS

In this topical review, we discussed the merits and challenges of current metabolic neuromonitoring technology used in the NICU to assess progression of HIE. The most valuable prognostic information following HIE is currently obtained from ^1^H MRS‐measured thalamic Lac/NAA, which can predict outcome at 2 years of age, but this gives us snapshot information and is performed after completion of therapeutic hypothermia. Therefore, we have looked to new optical technologies that have the potential to provide real‐time, non‐invasive, cot side measurements of metabolism immediately after birth.

Clinicians with the support of engineers and physicists have been seeking a cot side measurement that will allow identification of injury severity and prognostication of sick newborns in the neonatal intensive care unit for diagnosis. This ultimate indicator of brain tissue well‐being is unlikely to be a single measurement, but rather a biomarker created by a collage of several measurements with a brain tissue metabolic indicator as the lead marker. Optical technologies have the capacity to deliver a neuromonitoring platform at the cot side that can deliver in real time this collage of brain measurements that include brain oxygenation, blood flow and metabolism putting us closer to brain tissue well‐being indicator.

### Footnote

This Topical Review was invited following a keynote lecture “Monitoring with Near‐Infrared Spectroscopy Brain Oxygenation and Function" from Prof Ilias Tachtsidis at the 2019 meeting of the International Society on Oxygen Transport to Tissue.

## CONFLICT OF INTERESTS

The authors confirm that there are no conflicts of interest.

## AUTHOR CONTRIBUTIONS

GB completed the literature review and drafted the first version of the manuscript. GB, SM and IT contributed to the final version.

## ETHICAL STATEMENT

This paper reflects the authors’ impartial review of this topic. No ethical approval was required for this review.

Dr Bale is a Research Fellow in Medical Physics and Biomedical Engineering at University College London. Her work in the Biomedical Optics Research Laboratory focuses on developing non‐invasive, optical brain monitoring techniques for the measurement of cerebral oxygenation and metabolism in‐vivo.

Dr Mitra is a Wellcome Trust Research Fellow in Neonatal Neuroscinece in the Institute for Women's Health at University College London (UCL) and as a Consultant Neonatologist in University College London Hospital (UCLH). Dr Mitra has a keen interest in neonatal neuromonitoring and neuroprotection particularly in early identification and prognostication of perinatal brain injury to improve outcome.

Dr Tachtsidis is a Professor in Biomedical Engineering at University College London (UCL) in the UK. He is a senior member of the Biomedical Optics Research Laboratory and heads the Multi‐Modal Spectroscopy Group. Dr Tachtsidis has authored and co‐authored more than 100 research articles in the field of biomedical optics and NIRS.
